# Simvastatin-induced neuroprotective effect after brain injury is mediated by mitochondrial protection through modulation of the 18 ​kDa translocator protein

**DOI:** 10.1016/j.neurot.2025.e00803

**Published:** 2025-11-20

**Authors:** Reem Sakas, Tom Fishboom, Aviv Ben-Menashe, Yaseen Awad-Igbaria, Rana Nasra, Abraham O. Samson, Eilam Palzur, Jean F. Soustiel

**Affiliations:** a Azrieli Faculty of Medicine, Bar-Ilan University, Zefat, Israel; b Research Institute of Galilee Medical Center, Nahariya, Israel; c Department of Neurosurgery, Galilee Medical Center, Nahariya, Israel; d Department of Pathology, Galilee Medical Center, Nahariya, Israel

**Keywords:** Traumatic brain injury, Secondary brain injury, Translocator protein, Neuroprotection, Mitochondrial respiration

## Abstract

Traumatic brain injury (TBI) remains a leading cause of mortality and chronic disability. Among therapeutic agents investigated, simvastatin has emerged as a potential therapeutic opportunity for management of TBI although its mechanism of action has not been yet elucidated. Recent advance in 3D molecular docking simulations has suggested a possible interaction between simvastatin and the 18 ​kDa translocator protein (TSPO). Accumulating evidence suggest that The TSPO may play a pivotal role following TBI with TSPO ligands enhancing mitochondrial function and survival. Here, we examined the neuroprotective effects of simvastatin on cognitive and locomotor functions, histopathological outcome, and mitochondrial respiration following TBI in rats. In addition, molecular docking/interactions of simvastatin with TSPO were simulated. The current result show that simvastatin treatment significantly improved cognitive recovery in Morris water maze, motor performance in rotarod test, and neuronal density in the lesion area and hippocampus compared with untreated TBI groups. Importantly, these effects were attenuated by PK11195 pretreatment. Moreover, molecular docking simulations revealed that simvastatin exhibits a high binding affinity to TSPO, suggesting that its beneficial role could be the result of TSPO modulation. Furthermore, simvastatin treatment restored mitochondrial respiration by enhancing oxygen consumption rates across various respiratory states. In contrast, comparative analyses revealed that PK11195 attenuated simvastatin-induced respiratory enhancement, providing strong evidence for a TSPO-mediated mechanism of action of simvastatin. In conclusion, the current result highlights simvastatin's therapeutic potential in mitigating mitochondrial dysfunction and promoting neuroprotection effects following TBI. Our findings underscore mitochondrial protection as an important therapeutic target in TBI.

## Introduction

Traumatic brain injury (TBI) is the leading cause of mortality and severe morbidity in people under 45 years of age in Western industrialized countries [1]. TBI affects both young and adult individuals in their most productive years of life [2]. Yet, even with the best medical treatment and rehabilitation programs to improve life quality after brain injury, patients often experience only partial recovery and remain chronically disabled [3]. This remarkable challenge has prompted a continuous attempt to develop therapeutic means that can alleviate the consequences of TBI. Several pharmaceutical compounds have been investigated, showing promising results in the experimental setting, though none have shown any significant benefit in clinical trials [4].

TBI can lead to a wide range of long-term neurological and psychiatric disorders that persist well beyond the acute recovery phase [2]. The most common complication is post-traumatic headache (PTH), which can resemble migraine or tension-type headache and significantly impact quality of life [5]. Cognitive dysfunction, including memory deficits, impaired attention, and executive dysfunction, is also frequently observed [6]. In addition, motor impairments, such as weakness, poor coordination, and spasticity, may occur depending on the injury location and severity [7]. Psychiatric conditions such as depression, anxiety, post-traumatic stress disorder (PTSD), and emotional dysregulation are highly prevalent in TBI survivors [8]. These complications can worsen over time and contribute to social withdrawal, reduced independence, and long-term disability.

Brain injury-induced damage can be divided into two separate phases. The primary stage consists of immediate physical consequences of the injury itself and, as such, is irreversible and often fatal [9]. It can at best be prevented by prophylactic measures such as the use of a seatbelt or a helmet [10]. In contrast, the secondary phase of brain injury-induced damage represents the consequence of multiple interconnected cellular and subcellular events including, neuronal stress, energy deficit, which may eventually lead to the death of potentially viable cells [11]. Notably, the neuroinflammatory response—characterized by astrogliosis, microgliosis, and increased release of proinflammatory cytokines—is considered a major event during the secondary phase of injury [12]. The extent and clinical significance of secondary brain damage have been emphasized by Reilly et al. who showed that most head-injured patients who eventually died during their illness were, in fact, conscious at some time following the injury [13]. These findings suggest that secondary brain damage plays a major role in determining fatal outcomes, potentially underestimating the importance of preventive strategies targeting primary injury [9].

Among the various cellular structures targeted by TBI, the mitochondria have emerged as a pivotal junction where numerous harmful signals converge, ultimately leading to functional and structural impairments incompatible with cellular survival [9,14]. Efforts to elucidate the underlying mechanisms responsible for mitochondrial dysfunction have highlighted several critical elements within the mitochondrial architecture, which could serve as promising candidates for therapeutic investigations [15]. The mitochondrion is a unique organelle whose primary and crucial function is ATP production, the cell's energy currency, by utilizing a chemical concentration cascade within and across a two-fold membrane [16]. The outer mitochondrial membrane is semipermeable, allowing for the transit of small molecules [16]. In contrast, the inner membrane is impermeable, maintaining the critical gradient of protons created by the electron transport chain and crucial for activation of the ATP synthase [17]. Previous evidence suggests that the disruption of mitochondria may contribute to the damage and inflammation that occurs in the brain after the injury [18]. Furthermore, a substantial body of evidence suggests that functional and/or structural impairment of the mitochondria may result in induction of the mitochondrial permeability transition pore (mPTP) [19], leading to energy crisis and cell death [20].

Induction of mPTP results in an abrupt loss of inner mitochondrial membrane integrity, allowing water and small molecules to enter the mitochondria, leading to swelling and rupture of the outer mitochondrial membrane [21]. Although the precise trigger and mechanism behind mPTP induction are not fully understood, factors contributing to this process include oxidative stress, calcium overload, protein damage, and genetic mutations [22]. The mPTP induction causes the loss of mitochondrial membrane integrity and dissipation of the transmembrane potential (ΔΨm), which results in ATP synthase deficiency [23]. This cornerstone observation draws attention to the mitochondria's crucial role in the research effort for the therapeutic approach achievement in TBI [14].

Simvastatin, a statin commonly used to manage hypercholesterolemia [24], exerts its primary effect by inhibiting HMG-CoA reductase, the key enzyme responsible for cholesterol biosynthesis [25]. In addition, simvastatin influences the nitric oxide synthase system, displaying anti-inflammatory as well as antioxidative effects [26]. Additionally, it lowers intramitochondrial ionized calcium and subsequent oxidative stress while preventing mPTP induction and cytochrome *c* release [27]. Accordingly, it was hypothesized that these diverse properties may attribute to the drug an additional beneficial effect that may justify its possible implementation in the management of neurological disorders [28]. Interestingly, most of these presumed mechanisms of action share in common a mitochondrial protective end-effect. In the absence of a clear mitochondrial mechanism of action of simvastatin and in the search for a possible mitochondrial target of the drug that may explain its mitochondrial protective effects, we performed preliminary molecular docking simulations with outer membrane mitochondrial constituents (unpublished data) suggesting a high binding affinity of simvastatin to the 18 ​kDa translocator protein (TSPO). Located on the outer mitochondrial membrane, the TSPO has gained increasing attention for its potential role in mitochondrial function, especially in regulating the mPTP induction [29,30]. Under normal conditions, TSPO is expressed at low levels in the central nervous system but is upregulated in response to injury and inflammation [31]. Notably, an increase in TSPO expression has been observed in a preclinical model of TBI [32]. Given its role in mitochondrial health, TSPO has been proposed as a key therapeutic target for nerve injury conditions including TBI and neuropathic pain [[33], [34], [35]]. As such, the hypothesis that simvastatin acts as a TSPO ligand may explain some beneficial effects of the drug in the context of TBI. However, despite encouraging evidence from rodent models TBI showing robust neuroprotective benefits [[36], [37], [38], [39]], not all research involving simvastatin has yielded positive results. For instance, Chen et al. have shown the advantages of simvastatin in reducing edema in the Fluid Percussion Injury model, but they did not identify any therapeutic benefits in terms of recovering motor deficits [40]. In parallel, low-dose simvastatin treatment (5 ​mg/kg) produced modest improvements in motor function following TBI but failed to elicit significant cognitive or neuropathological recovery [25]. Yet, the relatively low dosage of simvastatin used in these models may be subject to criticism when the high metabolism of rodents is taken into consideration [41]. Accordingly, the principal aim of the present study was to investigate the effect of a higher dose of simvastatin (20 ​mg/kg) on cognitive, locomotor function, and histopathological outcomes following TBI. Furthermore, we attempted to characterize the effects of simvastatin on mitochondrial function while investigating a potential interaction with TSPO as a mechanism underlying these effects.

## Materials and methods

Male Sprague-Dawley rats (age 10 weeks, weight 250–300g) were used in the current study. All animal studies were conducted under the principles and procedures outlined in the Bar-Ilan University Research Institute in the Galilee Medical Center and were approved by the Institutional Animal Ethical Committee.

### Traumatic brain injury model

The current TBI model is based on a modified controlled cortical impact described by Palzur 2021(29), and Sakas 2023 [42]. Briefly, rats were anesthetized using 4 ​% isoflurane in 100 ​% oxygen within an induction chamber. After the induction of deep anesthesia to prevent pain responses, animals were fixed in a stereotaxic rat frame (Stoelting, Wood Dale, IL, USA), whereas they were kept under anesthesia over a nose cone with 2 ​% isoflurane using the SomnoSuite anesthesia delivery system (Kent Scientific, Torrington, CT, USA). Isothermal pads were attached to maintain the rats’ body temperature at 37 ​°C. Following skull exposure, a 6 ​mm diameter craniotomy was performed on the left hemisphere, 6 ​mm lateral to the sagittal suture, and midway between the lambda and bregma sutures. TBI was induced via open dura using a stereotaxic impactor (Leica Biosystems, Wetziar, Germany), and the velocity of the impactor reached 5.0 ​m/s with a depth of 2.5 ​mm using the 5 ​mm diameter tip below the dura matter. Throughout the impact, anesthesia was turned off temporarily for a few seconds to permit a breath of pure oxygen before the injury, as a preventive measure against possible post-traumatic apnea. The scalp injury was then sutured with a 3-0 silk suture, and the rat was allowed to recover from anesthesia in an individual cage. Notably, the sham-operated animals underwent all surgical procedures, including anesthesia, craniotomy, and scalp closure, but without cortical impact.

### Animal grouping and treatment

**Fig. 1:**
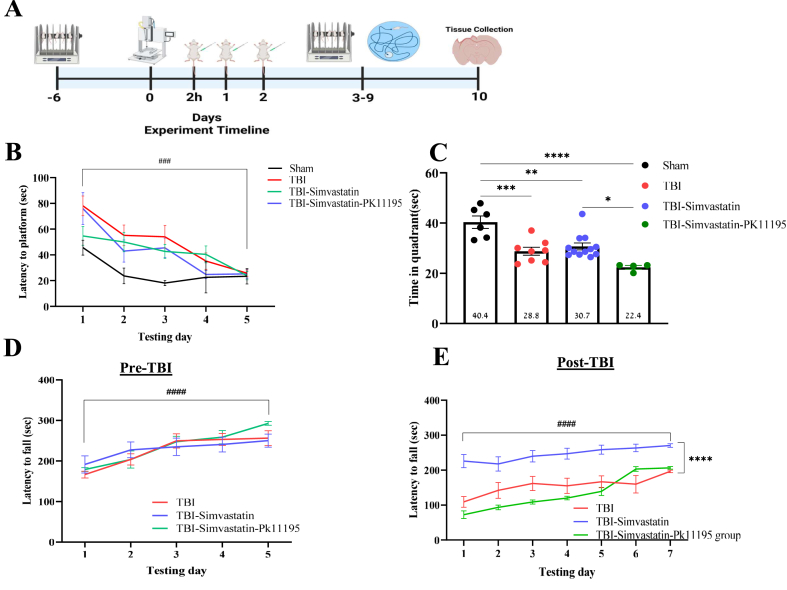
Simvastatin effects on cognitive and motor dysfunction following TBI damage. **(A)** The experimental timeline. Motor performances were measured before and after the TBI. After TBI, animals were randomly assigned to one of the experiment groups: TBI group. TBI-Simvastatin group. Treatment with Simvastatin was performed after 4h of the TBI, and twice a day for two days after TBI. Cognitive and motor performance were measured between day 3 and day 9 after the TBI. The animals were sacrificed on day 10 for immunohistochemistry purposes. (**B)** Spatial learning and memory performance in the Morris water maze test following TBI. The latency to reach the hidden platform was measured across training trials to assess learning performance. (**C)** Time spent in the quadrant in the probe trial in the absence of the platform on day 6 of the paradigm. (**D and E)** Motor performance in the rotarod test Pre-TBI **(D)**, and Post-TBI **(E)**. The latency to fall (seconds) was measured as an index of motor coordination and balance. (n ​= ​4–12) Mixed Model ANOVA; One way-ANOVA, followed by Tukey's. Mean ​± ​SEM. ^###^*P ​<* ​0.001 Compared to the 1^ST^ day. ∗*P ​<* ​0.05, ∗∗*P ​<* ​0.005, ∗∗∗*P ​<* ​0.001∗∗∗∗*P ​<* ​0.0001 Difference between groups.

Simvastatin and PK11195 were dissolved in ethanol (1 ​% of the final volume), and the stock solution was dissolved with physiological saline. The vehicle group was injected with saline solution (1 ​% ethanol of the final volume). All drugs were injected intraperitoneally (I.P). Following the injury, animals were allocated into two groups: 1- TBI-Vehicle group. 2- TBI-Simvastatin-treated group. Simvastatin injected at a dose of 20 ​mg/kg. Both groups received 5 injections, 4 ​h after the TBI and twice daily for 2 days post-injury (Fig. 1A). An additional experimental group was included to evaluate the therapeutic effect of simvastatin in combination with the TSPO ligand PK11195. Animals in this group underwent TBI induction followed by administration of simvastatin (20 ​mg/kg) and PK11195 (2 ​mg/kg), beginning 4 ​h post-injury and continuing twice daily for two consecutive days (five injections in total).

### Cognitive and locomotor functions

Animals were divided into TBI (n ​= ​12) and TBI ​+ ​Simvastatin (n ​= ​12) groups. Motor and cognitive performance were evaluated before and after injury using the rotarod and Morris water maze tests.

#### Morris water maze (MWM) test

MWM was used to evaluate spatial reference memory and involves placing rats in a circular pool filled with water containing a hidden platform [43]. A computer-controlled tracking system, Etho-Vision XT, monitors the rats' swim paths and time to find the platform over five days (i.e., from day 3–9 post-TBI). Four trials are conducted daily, assessing the rats' ability to learn and navigate to the platform, and measuring new spatial reference memory. On the sixth day, a probe test is conducted by removing the platform, allowing the rat to freely swim for 2 ​min in four trials, testing memory retention.

#### Rotarod locomotor test

Motor coordination and balance were assessed using rotarod. The apparatus consists of a rotating rod with machined grips. In test trials, the rotation speed increased from 4 to 40 ​rpm over 300 ​s. Each trial is terminated if an animal fell, clung, rotated for two complete rotations, or remained on for >300s. Latency to fall in seconds is automatically recorded for each trial. The experiment was conducted in three sessions per day for ten consecutive days before and after the TBI (i.e., from day 3–9 post-TBI). Before the TBI, rats were trained at a constant speed of 10 ​rpm on the Rotarod apparatus. The training trial continued until the rat could stay on the rod for 60 consecutive seconds without falling, turning around, or clinging to the rod [44].

### Histology

Following the end of the experiment on day 10, which likely corresponds to the subacute phase of TBI, a period characterized by the activation of secondary injury mechanisms—animals were deeply anesthetized, euthanized, and subsequently perfused transcranial with a heparinized saline solution containing 10 ​% sucrose in buffered saline, followed by 4 ​% buffered formaldehyde. Following this, the brain specimens were excised and immersed in a 4 ​% paraformaldehyde solution in phosphate buffer for 24 ​h, after that brain were embedded in paraffin. Subsequently, 5 ​μm thick sections were obtained from the coronal sections utilizing a microtome, with every fifth section being selectively preserved for analysis. The sections were then meticulously mounted onto adhesive glass slides procured from Matsunami. Separate cohorts were used for morphological and immunohistochemical analyses (n ​= ​5 per group).

### Immunohistochemistry

Immunostaining was performed using the Ventana Benchmark XT staining system (Ventana Medical Systems, Tucson, AZ, USA) with Anti-NeuN, clone A60, Monoclonal Antibody (1:50, Cat. # MAB377, Millipore corporations, Temecula, California). Hematoxylin and Eosin (H&E) staining was performed on tissue sections, involving a 5-min exposure to Lillie Mayer Alum's Hematoxylin solution for nuclear staining and up to 2 ​min with Eosin solution for cytoplasmic staining. The analyzed regions included the penumbra and sub-hippocampal areas.

### Microscopy

Microscopic examination was performed using the Eclipse Ci microscope (Nikon Corp., Japan), and digital images were acquired from regions of interest located adjacent to the lesion site. The first region, the perilesional cortex, was selected because it represents the primary site of impact and is directly affected by the initial mechanical trauma, thereby capturing the immediate structural damage associated with the acute phase of TBI. In contrast, the second region, the hippocampal subregions, was chosen due to its susceptibility to secondary injury processes, including inflammation, oxidative stress, and excitotoxicity, which typically develop during the subacute phase following the initial insult. At least 8 images were captured by the Eclipse Ci microscope (Nikon Corp., Kamogawa, Japan) with the same microscope settings and exposure time. Each section was analyzed to determine the average number of positively stained cells. Images were analyzed using Nikon NIS-Elements software and evaluated by an unbiased observer. The software was used to identify NeuN-positive stained cells, and the results were further confirmed through manual cell counting to ensure accuracy and reproducibility.

### Molecular docking

We examined the molecular interactions between the TSPO (PDB: 2MGY) and several recognized TSPO ligands, such as PK-11195 (CID: 1345), RO5-4864 (CID: 1688), Emapunil (CID: 6433109), Cholesterol (CID: 5997), and Simvastatin (CID: 54454), following the protocol previously described by Awad-Igbaria et al. [33]. Ligands library preparation was prepared using OpenBabel 3.0.1 [45]. Each ligand was optimized to ensure proper geometry and protonation states and saved as PDBQT format. Protein preparation and grid generation have been done using Autodock Tools 4 software. First, twenty conformations of TSPO were subjected to energy minimization, and water molecule removal and the protein structures were converted into the PDBQT format. For grid generation, a box of dimensions 25 ​× ​25 ​× ​25 ​Å^3^ (X, Y, Z) was defined for each potential binding site. Seven potential binding sites were identified based on a preliminary search using PK-1195 and RO5-4864 across all TSPO conformations. The coordinates for these binding sites were as follows: site 1: (−6.09,0.376,5.514), site 2: (13.72,3.55,7.14), site 3: (2.12,-10.49,8.4), site 4: (8.82,8.2,-3.5), site 5: (8.02,3.08,16), site 6: (−2,16.4,1.62), site 7: (1.63,-20.71,-8.8)), exhaustiveness ​= ​20, energy_range ​= ​3. Molecular docking simulations were performed using Autodock Vina [46,47], for each ligand across all conformations and binding sites identified above (7000 simulation in total). Each docking simulation was repeated ten times to ensure reproducibility and robustness of the results. Analysis and visualization were performed using PyMOL([48] and included ranking the binding poses based on their docking scores, docking site analysis. A 2D ligand interaction diagram was obtained using PoseEdit [[49], [50], [51], [52]].

### Mitochondrial function assessment

#### Mitochondria Isolation

Brain samples were harvested from the parietal region of the cerebral hemisphere of uninjured or injured rats, n ​= ​5 per group. The tissue samples were washed with ice cold (4 ​°C) mitochondrial respiration medium MiR05 (110 ​mM sucrose, 60 ​mM K ​+ ​-lactobionate, 0.5 ​mM EGTA, 3 ​mM MgCl2, 20 ​mM taurine, 10 ​mM KH2 PO4, 20 ​mM HEPES, 1 ​g/L BSA, adjusted to pH 7.1 with KOH). 200–250 ​mg of brain tissue was cut into small pieces and homogenized and centrifuged following the protocol described before [53,54]. Finally, the isolated mitochondria resuspended in MiR05.

#### O2k-FluoRespirometer

Mitochondrial respiration was assessed using the OROBOROS (Oxygraph-2k). The OROBOROS chambers were filled with 2.1 ​mL MiR05 and calibrated at 37 ​°C 30 ​min before the experiment. Oxygen concentration (μM) and oxygen flux per tissue mass (pmol O2/sec/mg) were recorded. The apparatus allows measurement at controlled oxygen levels, mitochondrial membrane potential, and ATP production. Moreover, this apparatus offers the capability to assess mitochondrial membrane potential simultaneously with mitochondrial respiration. The measurement of mitochondrial membrane potential was assessed using a cationic dye-safranin as was described before [34].

To investigate mitochondrial respiration states and electron transfer, we used the SUIT-020 Fluo mt D033 methodology [55]. The protocol evaluates mitochondrial respiration function and mitochondrial membrane potential under diverse conditions at the same time, including basal respiration and complex-linked substrates. The specific steps include a brain mitochondrial protein concentration of 0.25 ​mg/mL, following that we inject pyruvate (5 ​mmol/l), malate (5 ​mmol/l), ADP (2.5 ​mmol/l), and glutamate (2.5 ​mmol/l) for establishing the NADH pathway through complex I. Furthermore, succinate (10 ​mmol/l) was injected to assess the combined NADH and succinate pathways (maximal respiration) and subsequently introduce rotenone (1 ​μmol/l) inhibitor for complex I to evaluate the succinate pathway through complex II. Then we add oligomycin (2.5 ​μM) that represents a non-phosphorylation state after the activation of Succinate-CII and inhibiting ATP synthase to assess ATP-linked respiration, which is the OCR _(maximal respiration)_ minus the OCR _(oligomycin)_ [56]. Mitochondrial respiration is quantified as flux per mass (pmol O2/sec/mg). This approach provides valuable insights into mitochondrial function under various conditions, deepening our understanding of cellular respiration and mitochondrial membrane potential dynamics simultaneously. Mitochondrial membrane potential was measured according to the safranin fluorescence signal which we calibrated before starting the experiment (experimental concentration 2 ​μM), safranin is a lipophilic cationic dye, which allows us to assess its binding within the mitochondrial matrix, independent of delta psi investigation. The principle relies on the dye's fluorescence quenching as it binds to the negatively charged inner mitochondrial membrane [57].

### Data analysis

Statistical analyses were performed using IBM SPSS Statistics version 26 and GraphPad Prism. All results are presented as the Mean ​± ​SEM. Group comparisons were assessed using one-way ANOVA, while changes in cognitive locomotor tests were evaluated using mixed-model repeated-measures analysis of variance (GLM). Significant effects and interactions were further explored using Post hoc Tukey's test and independent-sample *t*-test. The accepted significance value was set at *P ​< ​0.05*.

## Results

### The effect of simvastatin treatment on cognitive dysfunction after TBI

To examine the combined effect of treatment and time-point interaction on cognitive performance, repeated measures analyses were used to compare performance in the five time-points (within-subject comparisons), and between the four groups (Sham, TBI, TBI- Simvastatin, TBI- Simvastatin-PK11195) as a between-subject variable. The analysis revealed a significant decrease in latency to reach platform in all groups over time (main effect of Time, F _(4,104)_ ​= ​20.580, P ​< ​0.001, η^2^ ​= ​0.442, Fig. 1B). As anticipated, TBI was associated in prolonged latencies to the platform in all TBI groups in comparison to sham animals (main effect of Group, F [1,26] ​= ​3.775, p ​= ​0.023, η^2^ ​= ​0.303, Fig. 1B). However, animals treated with simvastatin showed a faster recovery with significant shorter latency to reach the platform all days in comparison to the TBI group (Tukey's test p ​< ​0.05, Fig. 1B). Importantly, this protective cognitive effect of simvastatin proved to be attenuated by addition of PK11195 to the therapeutic regimen so that no significant difference could be noted between the control group and the simavastatin-PK11195 group (P ​= ​0.9361, Fig. 1B).

Yet, the rate of improvement across time was not significantly different between groups (Group ​× ​Time-points interaction, F _(4,104)_ ​= ​1.726, p ​= ​0.072, η^2^ ​= ​0.166, Fig. 1B). Following five consecutive days of training for acquisition, spatial accuracy was assessed by conducting a probe trial where the platform was removed from the pool. Substantial variations in the time spent in the quadrants were observed between groups (One-way ANOVA, F [3,29] ​= ​12.898, P ​< ​0.001, Fig. 1C). Further post hoc analysis revealed significant differences between the sham group compared to TBI, TBI-Simvastatin, TBI-Simvastatin-PK11195 groups (p ​< ​0.05, Fig. 1C), as well as between the TBI-Simvastatin group and the TBI-Simvastatin-PK11195 group (P ​= ​0.026, Fig. 1C).

### The effect of simvastatin treatment on the progression of motor impairment following TBI

Before the injury, there was no significant difference in motor performance (Latency to fall) between groups (main effect of Group, F [2,21] ​= ​0.082, P ​= ​0.921, η^2^ ​= ​0.008, Fig. 1D) and all groups showed similar motor performance in the rotarod test (Fig. 1D).). Following TBI and subsequent interventions treatment, repeated measures GLM analysis was used to compare performance across seven time-point (within-subject comparisons), and among the four groups (TBI, TBI–Simvastatin, TBI–Simvastatin–PK11195) as a between-subject factor. Following TBI, analysis revealed a significant main effect of time (F [6,60] ​= ​37.898, P ​< ​0.001, η^2^ ​= ​0.791) and group ​× ​Time-points interaction (F [6,60] ​= ​4.459, P ​< ​0.001, η^2^ ​= ​0.471), indicating distinct recovery dynamics between the different groups. Notably, TBI-simvastatin-treated animals exhibited superior motor performance across all time points in comparison to the TBI control group. As observed in the cognitive assessment, this beneficial locomotor effect of simvastatin was obliterated by PK11195. This was supported by a robust main effect of group (F:[2,10] ​= ​47.772, P ​< ​0.001, η^2^ ​= ​0.905), with post hoc analysis confirming significantly higher performance level of the TBI-Simvastatin group (Tukey's test, P ​< ​0.05; Fig. 1E).

### Neuronal density in cortical and subcortical injured regions

**Fig. 2:**
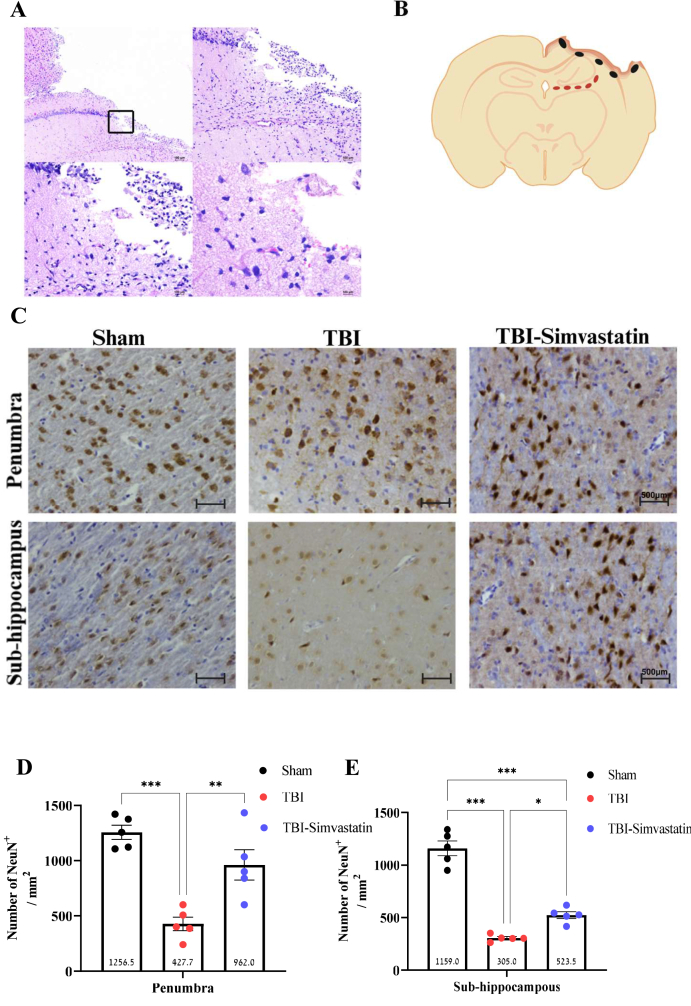
Cortical neuronal loss following TBI. **(A)** Morphological effects of TBI in brain tissue. Hematoxylin and eosin (H&E) stained sections, after TBI focusing on the penumbra site. Scale bar: 100 ​μm ​**(B)** illustration showing the process of counting positive cells in the two analyzed regions. **(C)** Cortical neuronal staining. Representative images of brain cortical neuronal staining with anti-NeuN for all groups in the penumbra and sub-hippocampus regions. Scale bar: 100 ​μm. **(D)** Number of positive neuronal cells/mm^2^ in the penumbra region. **(E)** Number of positive neuronal cells/mm^2^ in the sub-hippocampus region. *Scale bar:* 500 ​*μ*m. (n ​= ​5) Mean ​± ​SEM. One-way ANOVA was followed by Tukey's test. ∗P ​< ​0.05, ∖∗∗P ​< ​0.005, ∗∗∗P ​< ​0.001.

Immunohistochemical analysis was employed to evaluate neuronal integrity and lesion morphology following TBI. Hematoxylin-eosin staining confirmed the extent of cortical damage induced by the impact (Fig. 2A) that correlated with a marked reduction in neuronal density adjacent to the injury core, as expressed with NeuN staining. The regions selected for analysis within the neuronal areas of the sub-hippocampus and penumbra are illustrated in Fig. 2B. Quantification showed a significant neuronal loss in both the perilesional cortex and hippocampal subregions in the TBI and TBI-Simvastatin group in comparison with the sham group (one-way ANOVA, penumbra: F [2,14] ​= ​19.925, P ​< ​0.001; sub-hippocampus: F [2,14] ​= ​95.185, P ​< ​0.001; Fig. 2C, D, E). Simvastatin treatment significantly mitigated neuronal loss in both regions in comparison to the TBI group (P ​< ​0.05, Fig. 2C, D, E).

### Mitochondrial respiration following TBI and simvastatin treatment

**Fig. 3:**
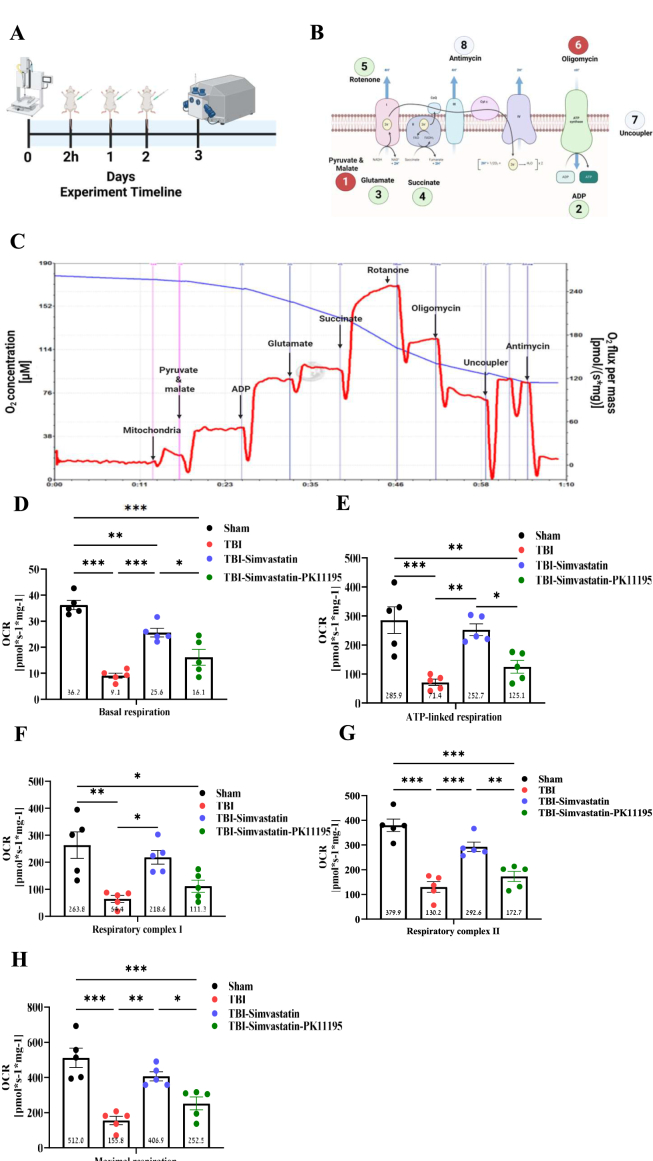
The effect of TBI on cortical mitochondrial oxygen consumption. **(A)** The experimental timeline. After TBI, animals were randomly assigned to one of the experiment groups: TBI group, TBI-Simvastatin group, TBI-Simvastatin-PK11195. Treatment with Simvastatin without/with PK11195 was performed after 4h of the TBI, and twice a day for two days after TBI. Mitochondrial function was examined on day 3 using O2k. **(B)** Protocol steps and respiratory state that were examined on isolated mitochondria from the injured hemisphere. The protocol examined mitochondrial respiration states at the LEAK-red circle, OXPHOS-green circle, and ET-gray circle. The circle number represents the order of stimulation/inhibition. **(C)** An illustration of the experiment in real-time is when oxygen consumption rates are measured in response to treatment with pyruvate-malate, glutamate, ADP, succinate, rotenone, oligomycin, CCCP, and antimycin. The red line illustrates the oxygen consumption rate, and the oxygen concentration is shown in the blue line. **(D)** Basal respiration state. (**E)** ATP-linked respiration was measured to reflect the efficiency of oxidative phosphorylation and mitochondrial energy production. **(F)** Mitochondrial respiration associated with complex I was assessed by adding pyruvate, malate, and glutamate substrates, followed by ADP stimulation. This step evaluates the contribution of the NADH-dependent electron transport chain to overall mitochondrial function. **(G)**. Mitochondrial respiration associated with complex II was determined by adding succinate after inhibiting complex I with rotenone. This allows for the evaluation of FADH2-dependent respiration and the integrity of the succinate dehydrogenase pathway. **(H)**. Maximal mitochondrial respiration capacity was measured following the addition of succinate, providing the evaluation of NADH-dependent electron & FADH2-dependent respiration. One-way ANOVA, followed with Tukey's test. (n ​= ​5) Mean ​± ​SEM. ∗*P* ​< ​0.05, ∗∗*P* ​< ​0.005, ∗∗∗*P* ​< ​0.0001.

To assess mitochondrial function post-injury, oxygen consumption rate (OCR) was measured in isolated brain mitochondria on day 3 following TBI as shown in Fig. 3A. The experimental protocol used is illustrated in Fig. 3B and C. Traumatic brain injury significantly impaired basal respiration compared to sham group (F [3,16] ​= ​34.48, P ​< ​0.001, Fig. 3D). Simvastatin treatment given to TBI rats restored basal OCR levels, showing a significant improvement over the TBI group (P ​< ​0.001) and TBI–Simvastatin–PK11195 group (P ​= ​0.0193), reaching values comparable to that of sham animals. ATP-linked respiration was also markedly reduced by TBI (F [3,16] ​= ​13.43, P ​< ​0.001, Fig. 3E). Noticeably, Simvastatin significantly enhanced ATP-linked OCR compared to the TBI group (P ​= ​0.0015) although this effect was hindered by addition of PK1195 to the therapeutic regimen (P ​= ​0.0239), with no significant difference with sham animals, indicating full functional recovery. Analysis of complex I-linked respiration revealed suppressed activity in the TBI group, and TBI–simvastatin–PK11195 group in comparison the sham group (F [3,16] ​= ​9.173, P ​< ​0.001, Fig. 3F), whereas Simvastatin significantly restored complex I activity compared to the TBI group (P ​= ​0.0122). Simvastatin also enhanced complex II-driven respiration compared to TBI (P ​< ​0.001) and TBI–Simvastatin–PK11195 (P ​= ​0.0062), again matching sham levels (F [3,16] ​= ​27.26, P ​< ​0.001, Fig. 3G). Maximal respiration (CI ​+ ​CII) was significantly impaired by TBI (F [3,16] ​= ​17.85, P ​< ​0.001; Fig. 3H). Yet, Simvastatin treatment robustly increased maximal OCR compared to both TBI (P ​= ​0.0012) and TBI–Simvastatin–PK11195 (P ​= ​0.0455), restoring function to the sham levels (Fig. 3H). Notably, the same experimental protocol used for the treated groups (Supplementary Fig. 1A–C) was also applied to sham animals, in which no differences in mitochondrial respiration were observed following chronic treatment with Simvastatin or Simvastatin–PK11195 (Supplementary Fig. 1D–H).

### The effect of simvastatin treatment on mitochondrial membrane potential following TBI

**Fig. 4:**
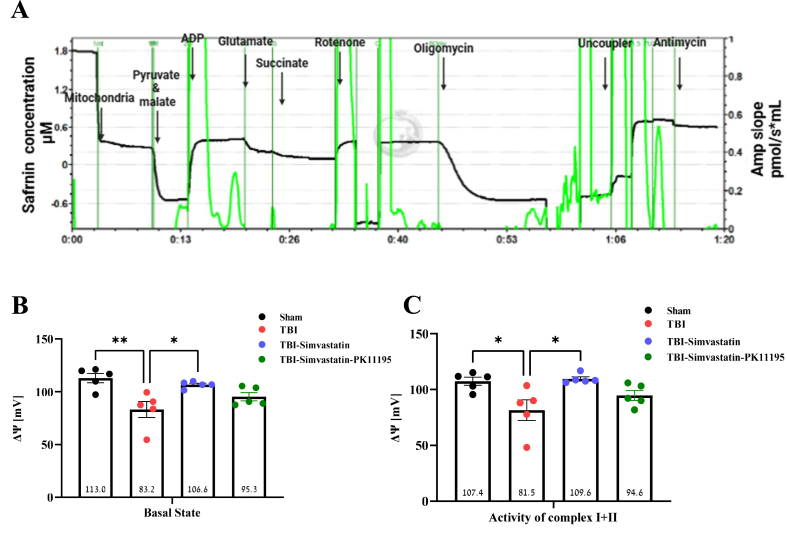
Mitochondrial membrane potential following TBI. **(A)** Representative trace showing changes in Safranin fluorescence. The black trace represents the Safranin fluorescence, which correlates with mitochondrial membrane potential, while the green trace indicates the AMP signal slope, reflecting changes in mitochondrial membrane potential activity, in response to treatment with pyruvate-malate, glutamate, ADP, succinate, rotenone, oligomycin, CCCP, and antimycin. **(B)** Basal mitochondrial membrane potential in isolated mitochondria from the injured hemisphere. (**C)** Mitochondrial membrane potential of the activity of complexes NADH-dependent electron CI and FADH2-dependent respiration CII. One-way Anova-Followed with Tukey's test. (n ​= ​5) Mean ​± ​SEM. ∗*P* ​< ​0.05, ∗∗*P* ​< ​0.005.

Mitochondrial membrane potential was assessed in isolated brain mitochondria on day 3 post-injury to evaluate mitochondrial integrity. The experimental protocol used is illustrated in Fig. 4A. TBI significantly reduced membrane potential in non-treated animals compared to sham (F [3,16] ​= ​7.315, P ​= ​0.0026, Fig. 4B). In contrast, Simvastatin treatment restored membrane potential relative to the TBI group (P ​= ​0.0168), with values similar to sham. Interestingly, PK11195 addition markedly attenuated this effect although the difference did not reach statistical significance. Under substrate conditions supporting Complex I and II activity, TBI also led to a significant reduction in membrane potential compared to sham, TBI–simvastatin, and TBI–simvastatin–PK11195 groups (F [3,16] ​= ​5.514, P ​= ​0.0085; Fig. 4C). Simvastatin significantly improved membrane potential compared to TBI (P ​= ​0.0118), with no significant difference from sham. PK11195 co-administration did not alter membrane potential relative to sham or TBI-simvastatin group. Notable, no effects were observed in mitochondrial membrane potential following chronic treatment with Simvastatin or Simvastatin-PK11195 in sham animals (Supplementary Fig. 2A–C).

### Comparative molecular docking analysis of TSPO ligands and simvastatin

**Fig. 5:**
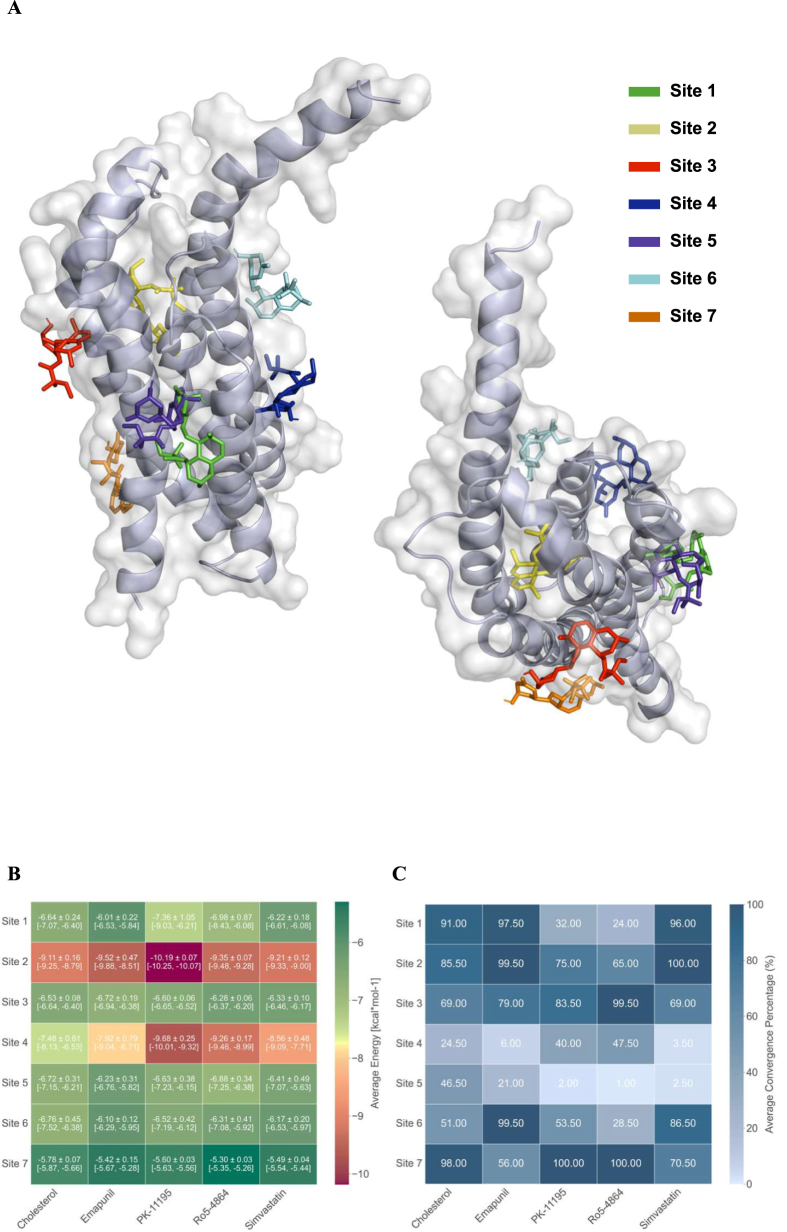
Predicted binding characteristics and simulation convergence of known TSPO ligands and Simvastatin across potential binding sites of TSPO. **(A)** Schematic representation illustrating the location of seven potential ligand binding sites (numbered 1–7) identified within the structure of TSPO. **(B)** A heatmap displaying the percentage of simulation convergence for Simvastatin, Emapunil, Ro5-4864, PK-11195, and cholesterol over the seven possible binding sites. Each square shows the percentage of simulations in which the ligand stayed consistently linked to the corresponding binding site (n ​= ​200 total per ligand). Greater ligand specificity and stability for that specific site are suggested by darker colors, which also show a larger percentage of convergence. **(C)** Heatmap showing the average estimated binding energy (kcal/mol) for Simvastatin and different TSPO ligands across the seven binding sites that have been found. The average binding energy ​± ​standard deviation and the minimum and maximum average binding energy of 20 different TSPO conformations (each averaged from 10 independent simulations) are presented in each square. The color intensity indicates the amount of the average binding energy, with warmer colors suggesting better (lower) binding energies. All these findings point to Site 2 as having the most promising binding properties among the seven suggested binding sites; this conclusion is in line with previous research on TSPO ligand binding.

To test the hypothesis that simvastatin may act as a TSPO ligand, we employed molecular docking simulations across seven possible binding sites (Fig. 5A). Comparative analysis (Fig. 5B,C) revealed distinct binding profiles for each ligand. PK-11195 exhibits a high binding affinity at site 2 (−10.19 ​± ​0.07 ​kcal/mol) with moderate convergence (75 ​%), and complete convergence at site 7 (100 ​%), but a lower binding energy of −5.60 ​± ​0.03 ​kcal/mol. Ro5-4864, a known TSPO agonist, demonstrated high convergence at Sites 3 (99.5 ​%) and 7 (100 ​%), with binding energies of −6.28 ​± ​0.06 ​kcal/mol and −5.30 ​± ​0.03 ​kcal/mol, respectively. Simvastatin displayed high convergence at sites 1 (96 ​%), 2 (100 ​%), and 6 (86.5 ​%), with a binding energy of −9.21 ​± ​0.12 ​kcal/mol at Site 2, but lower binding energies at sites 1 and 6 (−6.22 ​± ​0.18 ​kcal/mol and −6.17 ​± ​0.20 ​kcal/mol, respectively). Emapunil, like simvastatin, showed strong convergence (99.5 ​%) at site 2, along with a high average binding energy of −9.52 ​± ​0.47 ​kcal/mol, and at site 6 (99.5 ​%) but a lower binding energy of −5.42 ​± ​0.15 ​kcal/mol. Cholesterol's highest average binding energy was at site 2 (−9.11 ​± ​0.16 ​kcal/mol), with moderate convergence (85 ​%). The combination of simvastatin's 100 ​% convergence at site 2, a favorable binding energy comparable to other TSPO ligands, and the overall preference of known TSPO ligands for this site suggests that site 2 is the most promising target for simvastatin. Therefore, our analysis focused on this site.

### Simvastatin affinity for PK11195 binding pocket

**Fig. 6:**
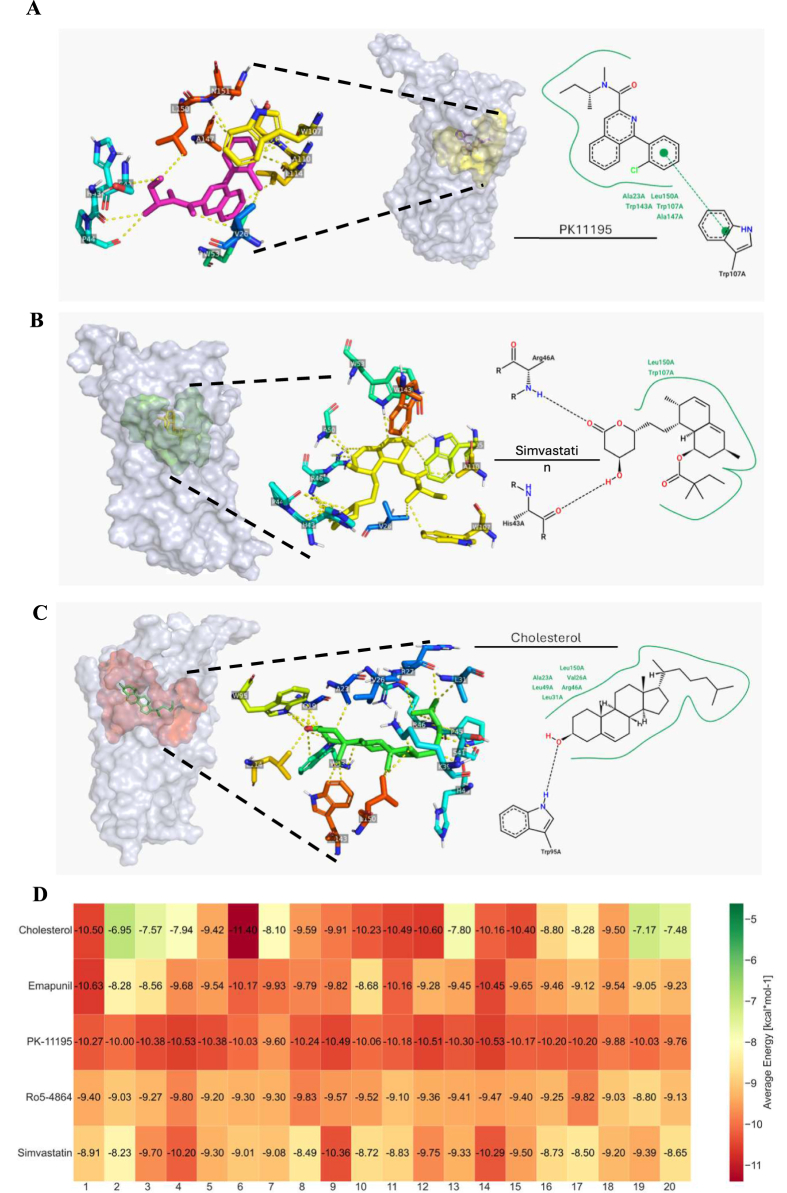
Simvastatin's potential interactions within the Translocator Protein (TSPO) site 2 compared to known TSPO ligands and their predicted binding affinities. **(A)** PK-11195 binding diagram at site 2, emphasizing important intermolecular interactions: pi interactions (cyan dashed lines) and hydrophobic contacts (green arcs). PoseEdit and PyMOL were used for the visualization. **(B)** Simvastatin's predicted binding position inside TSPO site 2 displays possible hydrophobic interactions (green arcs) and hydrogen bonds (blue dashed lines). **(C)** Illustrative binding position of endogenous cholesterol at the TSPO binding site 2: hydrophobic contacts (green arcs) and hydrogen bonds (blue dashed lines). **(D)** Heatmap showing the average estimated binding energy (n ​= ​10 separate dockings in each conformation) of Simvastatin, Cholesterol, PK-11195, Emapunil, and RO5-4864 over twenty TSPO structural states. Warmer colors in the heatmap indicate a more favorable (lower) average binding energy, implying a higher anticipated binding affinity.

Consistent with Hien T.T. Lai [58] findings, PK11195 was observed to bind within, site 2, the pocket formed by TSPO TM1, TM2, and TM5 alpha helices. Our molecular docking simulations suggest that residues Ala23, Val26, His43, Trp107, Trp143, Ala147, and Leu150 play a role in stabilizing PK11195 (Fig. 6A). Simvastatin also exhibited significant binding affinity to TSPO, with an average binding energy of −9.21 ​± ​0.12 ​kcal/mol across all conformations and converged to the same binding site in 100 ​% of simulations (200/200 runs), indicating high reproducibility. simvastatin's maximal binding energy was observed with TSPO conformation 4 (−10.20 ​kcal/mol), 9 (−10.36 ​kcal/mol), and 14 (−10.29 ​kcal/mol). Residues involved in simvastatin binding included Val23, His43, Arg46, Trp53, Trp107, and Trp143 (Fig. 6B). Comparative analysis showed that simvastatin had a higher binding affinity than cholesterol in 50 ​% of conformations, Ro5-4864 in 45 ​%, and Emapunil in 25 ​%, but did not surpass PK11195 in any conformation. As previously described, Emapunil displayed strong convergence and high binding energy at site 2, while cholesterol showed its highest average binding energy at site 2 with moderate convergence (Fig. 6C).

. The average binding affinity of PK11195 across all TSPO conformations was −10.19 ​± ​0.07 ​kcal/mol, with the highest affinity observed in conformations 4 and 14 (−10.53 ​kcal/mol at both (Fig. 6D).

## Discussion

Pursuing a potential therapeutic intervention for TBI has persisted over several decades [59]. However, translating promising experimental findings into successful clinical trials has repeatedly failed during the past three decades [60]. Recently, simvastatin, a widely prescribed lipid-lowering medication, has gained attention beyond its cholesterol-lowering effects as a potential therapeutic modality. Yet, despite encouraging results obtained both in the pre-clinical [61]and clinical [62], settings, the actual beneficial effect of simvastatin in TBI has not been clearly established. Here, we found that simvastatin administered at a dose of 20 ​mg/kg resulted in improved functional outcomes. Among the various deleterious consequences of TBI, cognitive dysfunction is a prevalent sequel, significantly impairing the quality of life of head-injured patients [6]. In the present settings, simvastatin proved to significantly improved cognitive outcome following TBI, as evidenced by a faster recovery in latency to the platform in the MWM test. While all groups demonstrated a significant reduction in latency over time, the simvastatin-treated animals showed a faster recovery compared to the TBI control group. These results suggest that the neuroprotective effect mediated by simvastatin eventually supports functional recovery after TBI.

Motor impairment is another common consequence of TBI, affecting mobility and overall independence [7]. In our study, simvastatin also demonstrated significant benefits in motor coordination, as measured by the rotarod test. While no differences were observed in motor performance before the injury, simvastatin-treated animals showed improved recovery over time compared to that of the control group. These findings align with prior research suggesting the potential of simvastatin to exert positive effects on sensorimotor function [41,63,64].

Although experimental evidence for various possible pathways of neuroprotection have been provided including antioxidative, anti-inflammatory effects, and regulation of progenitor cells [65], the actual mechanism of action of simvastatin remains elusive. A pivotal event in the progression of the secondary injury is the dysfunction of mitochondria, responsible for cellular energy production and homeostasis [66]. This dysfunction compromises the selective permeability of mitochondrial membranes, culminating in the induction of the mPTP, dissipation of the mitochondrial membrane potential, release of pro-apoptotic molecules, energy crisis and ultimately cell death [67,68]. Interestingly, however, the observation of delayed neurocognitive improvement in spatial learning mediated by simvastatin treatment in a rodent model of TBI has raised the hypothesis that statins may affect apoptosis [69]. This theory was later supported by the findings of an additional study showing a significant upregulation of Bcl-2 in simvastatin-treated Guinea [70], presumably mitigating the consequence of release of mitochondrial pro-apoptotic molecules. In this regard, the current study provides direct evidence that treatment with simvastatin does in fact alleviate TBI-induced mitochondrial dysfunction, as evidenced by improved mitochondrial respiration. Specifically, simvastatin revealed a significant improvement in basal respiration, ATP-Linked respiration, complex I-linked and complex II-linked oxygen consumption following TBI. These findings indicate a potential protective role of simvastatin in preserving mitochondrial function and enhancing ATP production, both of which are critical for cellular survival and recovery, particularly in the context of TBI. The enhancement of basal respiration and oxygen consumption through complex I and complex II pathways align with the notion that simvastatin may improve mitochondrial bioenergetics and are in accordance with previously reported findings [71]. Consequently, it can be expected that preservation of mitochondrial respiration and capacity to maintain a sufficient transmembrane potential may prevent mPTP induction and therefore mitigate TBI-induced mitochondrial damage as previously suggested [72]. Furthermore, simvastatin was found to increase ATP production, while TBI resulted in a reduction. This is reflected in the ATP-linked respiration results, which assess the ability of cells to meet their energy requirements and survive the process of secondary brain damage. Together, our findings of mitochondrial protection and enhanced function suggest that simvastatin's positive impact on mitochondrial respiration may eventually promote neuronal survival as confirmed by the increased number of living neurons in both the penumbra and the sub-hippocampus regions of the brain in comparison with animals of the control group and by earlier studies [69].

However, no clear mitochondrial mechanism of action of simvastatin has been yet described to explain this mitochondrial protective effect. Based on the three-dimensional morphology to the simvastatin molecule, we performed preliminary molecular docking simulations with outer membrane mitochondrial constituents (unpublished data) suggesting the possibility of a high binding affinity of simvastatin to the 18 ​kDa translocator protein (TSPO). The TSPO has been implicated in various cellular functions, including cholesterol transport [73], steroidogenesis [74], and modulation of reactive oxygen species production [75,76]. Although scarcely present in the normal brain tissue under physiological conditions, TSPO has been shown to be upregulated in response to a wide array of neuropathological conditions, such as cancer [77], neuroinflammation [78], neuropathic pain [32], and traumatic brain injury [79]. Initially isolated as a co-precipitate with the voltage-dependent anion channel and the adenine nucleotide translocator, both believed in time to be constituents of the mPTP, it was hypothesized that TSPO may act a modulator of the mPTP. Numerous studies have since then provided accumulating evidence showing that TSPO lignads might mediate neuroprotective effects by modulating mPTP activity [[80], [81], [82]].

Indeed, the results of the molecular docking study are theoretically supporting our initial assumption that simvastatin may produce a mitochondrial protective effect by directly modulating TSPO activity as a TSPO ligand. Our findings showed that simvastatin did exhibit a reasonable binding affinity to TSPO expressed by a high binding energy across all conformations and converged to the same binding site in all simulations. Further, comparative analysis showed that simvastatin had a higher binding affinity than cholesterol in 50 ​% of conformations, Ro5-4864 in 45 ​%, and Emapunil in 25 ​%, though did not surpass PK11195 in any conformation.

These bioinformatical observations are in accordance with mitochondrial respiratory assessments showing that addition of PK11195 to the mitochondrial pellets resulted in the obliteration of all the previously shown protective physiological effects mediated by simvastatin, presumably as the consequence of a binding competition between the two molecules at the TSPO level with higher affinity of PK11195 presumably inhibiting the binding of simvastatin to TSPO. Finally, the fact that co-treatment with PK11195 eventually prevented from simvastatin to mediate the same motor and cognitive beneficial effect observed in rats treated with simvastatin alone further supports the hypothesis of a neuroprotective effect of the drug mediated by TSPO modulation. Our molecular docking results suggest that Simvastatin may modulate TSPO activity through reasonable binding affinity; however, this remains theoretical, as direct binding or competitive displacement has not been demonstrated experimentally.

Several limitations of our study should be acknowledged. First, considering the size of each group was modest and using only male subjects may influence the interpretation of the findings. Therefore, replications among larger samples, including females, are critical to establish the validity of the current findings and infer their practical implications. While we included appropriate control and experimental groups, additional conditions—such as TBI treated with PK11195—remain to be examined in order to further clarify the mechanism by which PK11195 obliterated to effect yielded by simvastatin. Yet, although the question of a TSPO-independent deleterious effect of PK11195 explaining this effect is of concern, we were able to show in a previous study that neither the mitochondrial function or the neuronal survival were significantly affected by PK11195 following traumatic brain injury (Ref: Soustiel et al. Exp. Neurol, 2008). Accordingly a putative hypothetical detrimental effect of PK11195 is unlikely to account for the obliteration of the simvastatin effect by PK11195 and therefore did not seem to justify a much larger number of animals to answer such a question. Nevertheless, including an additional control group in future studies may further strengthen the overall interpretation of the findings. In addition, TSPO expression was not directly quantified in our experimental TBI model; future studies will include Western blot or IHC analyses to confirm TSPO upregulation and strengthen mechanistic interpretation. Furthermore, this study was conducted exclusively in a rodent model, without parallel validation in human cell-based systems. Given the well-documented differences in drug sensitivity between rodent and human neurons, as well as the limited translational success of many neuroprotective compounds from preclinical to clinical trials, the inclusion of basic in-vitro experiments using human neuronal cells would have significantly enhanced the translational relevance of our findings. Nevertheless, clinical trials are ultimately needed to validate the therapeutic potential of simvastatin in human TBI patients. In particular, future studies should carefully evaluate the optimal dosing and safety profile of simvastatin in the context of TBI to ensure both efficacy and tolerability. Despite these limitations, our findings highlight the significant potential of simvastatin as a therapeutic intervention in TBI. By improving locomotor outcomes, promoting neuronal survival, and preserving mitochondrial function, simvastatin offers a multi-faceted approach to mitigate the consequences of TBI. These findings hold promising implications for advancing TBI treatment strategies and offer hope for improving the lives of individuals affected by this devastating condition.

## Ethics approval and consent to participate

All animal procedures were approved by the Bar-Ilan University Animal Care Committee (approval number 2201-108-4) and were carried out by the National Institutes of Health Guide for the Care and Use of Laboratory Animals.

## Consent for publication

All authors mentioned agreed to the publication of the manuscript.

## Availability of data and materials

All data from this study is included in the manuscript.

## Author contributions

R.S. performed all experiments, interpreted the result, and wrote the initial manuscript draft. A.B.M. assisted with the mitochondrial respiration data collection and analysis. T.F. assisted with Molecular docking data and analysis. Y.A.I. undertook the statistical analysis and edited the final manuscript. J.F.S. assisted with methodology, project administration, and resources. E.P. and Y.A.I. conceived, designed, and supervised the research, provided technical and interpretational advice, and edited the final manuscript. All the authors contributed to the discussion and have approved the final manuscript.

## Declaration of competing interest

The authors declare that they have no known competing financial interests or personal relationships that could have appeared to influence the work reported in this paper.
