# Supplementary material for: Simvastatin-induced neuroprotective effect after brain injury is mediated by mitochondrial protection through modulation of the 18 ​kDa translocator protein
Source: Neurotherapeutics. 2025 Nov 20;23(1):e00803. doi: 10.1016/j.neurot.2025.e00803 (PMC12976529; doi:10.1016/j.neurot.2025.e00803)
Supplement: Multimedia component 1 [file mmc1.docx]

**Simvastatin-induced Neuroprotective Effect after brain injury is mediated by mitochondrial protection through modulation of the 18 kDa Translocator Protein**

Reem Sakas^1,2†^, Tom Fishboom^1,2†^, Aviv Ben-Menashe^1,2^, Yaseen Awad-Igbaria^1,2^,

Rana Nasra^4^, Abraham O Samson^1^, Eilam Palzur^1,2†^, Jean F. Soustiel^1,2,3*†^

**Affiliations**

^1^Azrieli Faculty of Medicine, Bar-Ilan University; Zefat, Israel.

^2^ Research Institute of Galilee Medical Center, Nahariya, Israel.

^3^Department of Neurosurgery, Galilee Medical Center, Nahariya, Israel.

^4^Department of Pathology, Galilee Medical Center, Nahariya, Israel.

†These authors contributed equally to this work.

^*^Corresponding author:

[Jeans@gmc.gov.il](mailto:Jeans@gmc.gov.il)

Reemsakas@gmail.com

P.O.B 21 Nahariya 22100 Israel

Tel: +972-04-9107538

Fax: +972-04-9107061

This file includes:

1. Supplementary figures and legends:

Supplementary Figure. S1: The effect of Simvastatin and PK11195 on cortical mitochondrial oxygen consumption in Sham-operated rats.

Supplementary Figure. S2 Mitochondrial membrane potential following sham procedure.


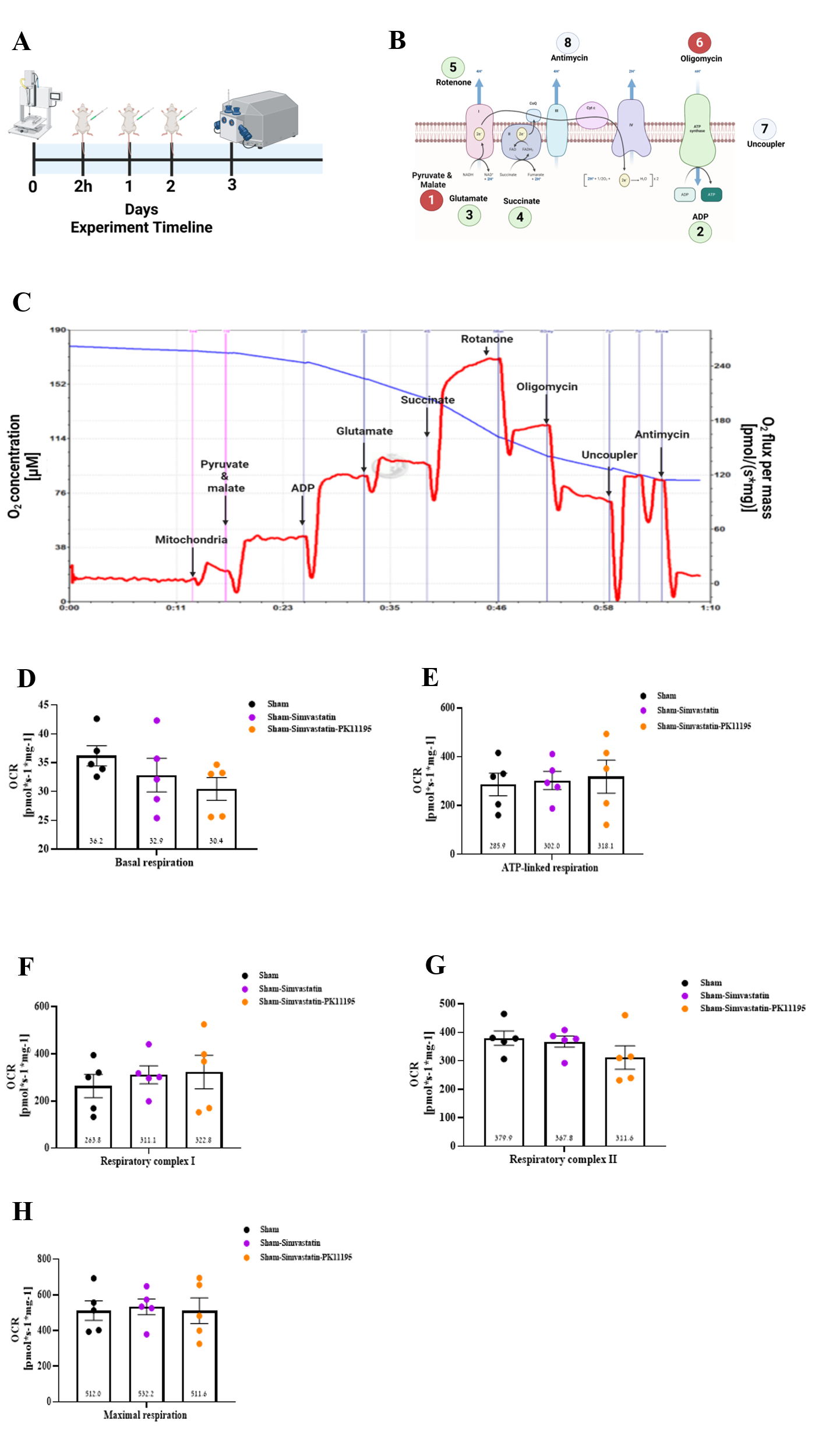


**Figure S1.** The effect of Simvastatin and PK11195 on cortical mitochondrial oxygen consumption in Sham-operated rats. **(A)** The experimental timeline. After sham surgery, animals were randomly assigned to one of the three experiment groups: Sham, Sham-Simvastatin, Sham-Simvastatin-PK11195. Treatment with Simvastatin without/with PK11195 was performed after 4h of the TBI, and twice a day for two days after TBI. Mitochondrial function was examined on day 3 using O2k. **(B)** Protocol steps and respiratory state that were examined on isolated mitochondria from the injured hemisphere. The protocol examined mitochondrial respiration states at the LEAK-red circle, OXPHOS-green circle, and ET-gray circle. The circle number represents the order of stimulation/inhibition. **(C)** An illustration of the experiment in real-time is when oxygen consumption rates are measured in response to treatment with pyruvate-malate, glutamate, ADP, succinate, rotenone, oligomycin, CCCP, and antimycin. The red line illustrates the oxygen consumption rate, and the oxygen concentration is shown in the blue line. **(D)** Basal respiration state. **(E)** ATP-linked respiration was measured to reflect the efficiency of oxidative phosphorylation and mitochondrial energy production. **(F)** Mitochondrial respiration associated with complex I was assessed by adding pyruvate, malate, and glutamate substrates, followed by ADP stimulation. This step evaluates the contribution of the NADH-dependent electron transport chain to overall mitochondrial function. (**G)**. Mitochondrial respiration associated with complex II was determined by adding succinate after inhibiting complex I with rotenone. This allows for the evaluation of FADH2-dependent respiration and the integrity of the succinate dehydrogenase pathway. **(H).** Maximal mitochondrial respiration capacity was measured following the addition of succinate, providing the evaluation of NADH-dependent electron & FADH2-dependent respiration. One-way ANOVA, followed with Tukey’s test. (n=5) Mean ± SEM. *P<0.05, **P<0.005, ***P<0.0001.


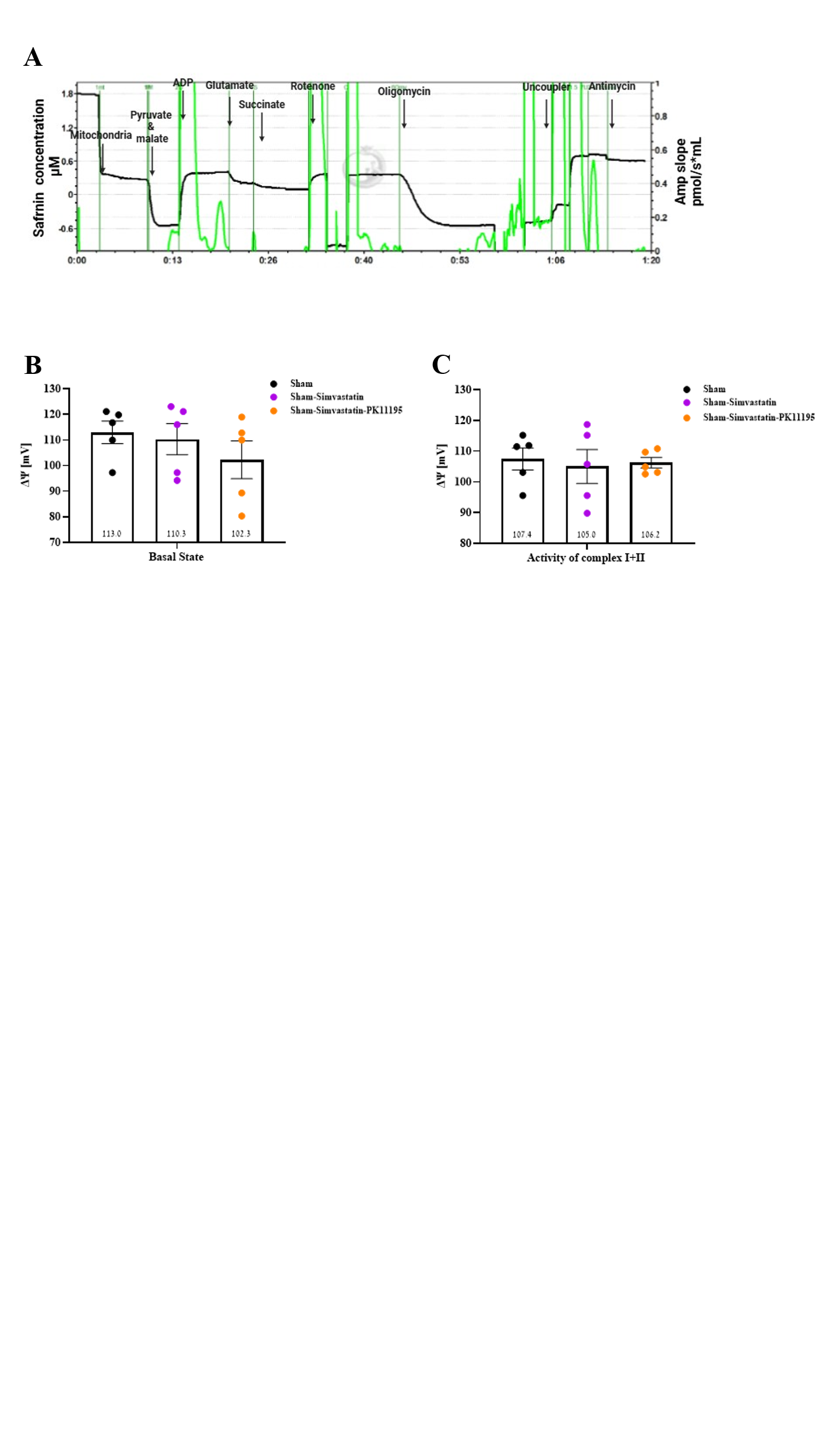


**Figure S2.** Mitochondrial membrane potential following sham procedure. **(A)** Representative trace showing changes in Safranin fluorescence. The black trace represents the Safranin fluorescence, which correlates with mitochondrial membrane potential, while the green trace indicates the AMP signal slope, reflecting changes in mitochondrial membrane potential activity, in response to treatment with pyruvate-malate, glutamate, ADP, succinate, rotenone, oligomycin, CCCP, and antimycin. **(B)** Basal mitochondrial membrane potential in isolated mitochondria from the injured hemisphere. (**C)** Mitochondrial membrane potential of the activity of complexes NADH-dependent electron CI and FADH2-dependent respiration CII. One-way Anova-Followed with Tukey’s test. (n=5) Mean ± SEM. ^*^*P*<0.05, ^**^*P*<0.005.
